# Security Evaluation of Provably Secure ECC-Based Anonymous Authentication and Key Agreement Scheme for IoT

**DOI:** 10.3390/s25010237

**Published:** 2025-01-03

**Authors:** Kisung Park, Myeonghyun Kim, Youngho Park

**Affiliations:** 1 Department of Computer Engineering (Smart Security), Gachon University, Seongnam 13120, Republic of Korea; kisung@gachon.ac.kr; 2 System Security Research Section, Electronics and Telecommunications Research Institute, Daejeon 34129, Republic of Korea; kimmh12@etri.re.kr; 3 School of Electronic and Electrical Engineering, Kyungpook National University, Daegu 41566, Republic of Korea

**Keywords:** security analysis, Internet of Things, security attacks, session key security

## Abstract

The proliferation of the Internet of Things (IoT) has worsened the challenge of maintaining data and user privacy. IoT end devices, often deployed in unsupervised environments and connected to open networks, are susceptible to physical tampering and various other security attacks. Thus, robust, efficient authentication and key agreement (AKA) protocols are essential to protect data privacy during exchanges between end devices and servers. The previous work in “Provably Secure ECC-Based Anonymous Authentication and Key Agreement for IoT” proposed a novel AKA scheme for secure IoT environments. They claimed their protocol offers comprehensive security features, guarding against numerous potential flaws while achieving session key security. However, this paper demonstrates through logical and mathematical analyses that the previous work is vulnerable to various attacks. We conducted a security analysis using the extended Canetti and Krawczyk (eCK) model, which is widely employed in security evaluations. This model considers scenarios where an attacker has complete control over the network, including the ability to intercept, modify, and delete messages, while also accounting for the potential exposure of ephemeral private keys. Furthermore, we show that their scheme fails to meet critical security requirements and relies on flawed security assumptions. We prove our findings using the automated validation of internet security protocols and applications, a widely recognized formal verification tool. To strengthen attack resilience, we propose several recommendations for the advancement of more robust and efficient AKA protocols specifically designed for IoT environments.

## 1. Introduction

The Internet of Things (IoT) is rapidly growing due to advancements in chipset production and embedding technologies. End devices, including sensors and actuators, are now ubiquitous in various fields, such as intelligent transportation, smart grids, healthcare, and intelligent manufacturing [1]. According to recent estimates, the number of IoT connections will reach 39.6 billion by 2033 [2]. In IoT applications, data security and user privacy are paramount concerns, particularly regarding sensitive information, such as consumption habits, locations, and communication activities [3,4]. “Authentication and key agreement” (AKA) schemes are widely adopted for secure mutual authentication and data privacy [5]. However, resource-limited end devices are often deployed in unattended environments and connected to open networks, which pose significant challenges in maintaining secure mutual AKA with servers [6,7].

In 2024, Hu et al. [8] presented a provably robust AKA scheme for IoT environments, claiming that it offers anonymity and high security. However, our analysis reveals that their scheme inadequately addresses physical capture attacks and fails to resist various security threats, including impersonation attacks. Its vulnerability arises from the risk of secret parameters kept in memory to be exposed to attackers through physical capture attacks.

### Motivations and Contributions

This work primarily aims to identify significant security vulnerabilities of the previous work scheme. We demonstrate that an attacker can easily compromise the session keys (SKs) between entities through impersonation attacks in this scheme. Consequently, their scheme fails to ensure SK security, as verified via a formal security analysis using the well-established “real-or-random” (ROR) model [9]. We also perform a formal simulation analysis using the “automated validation of internet security protocols and applications” (AVISPA Version 1.6) tool. These formal analysis methods are useful for analyzing protocol security [10,11,12,13]. Finally, we show that the scheme is unsuitable for real IoT environments and present recommendations to address these vulnerabilities and improve its security. IoT devices may be either mobile or fixed in one location, so they are relatively easy to capture. Additionally, embedded devices have resource-limited characteristics, which hinders the implementation of computationally intensive cryptographic methods. Therefore, attackers can easily gain control over compromised IoT devices. Robust security technologies must be in place to ensure the safety of IoT devices, even if they are captured.

The rest of this paper is structured as follows: Section 2 and Section 3 present a review of related works and the required preliminaries, respectively. Section 4 and Section 5 provide a review and cryptanalysis, respectively, of the previous work. Section 6 proposes solutions to improve security and defenses against potential attacks. Section 7 provides our conclusions and outlines directions for future research.

## 2. Related Works

Numerous IoT AKA schemes based on elliptic curve cryptosystems (ECCs) have been developed [14,15,16,17,18,19,20,21,22,23]. In 2020, Fang et al. [14] proposed an IoT AKA scheme that uses a trust model to deploy heterogeneous IoT smart devices. However, this scheme incurs higher operation and network costs and is vulnerable to ephemeral secret leakage (ESL) attacks [15]. ESL attacks occur when ephemeral secrets are compromised, allowing an adversary to obtain private keys and determine SKs from intercepted messages. Also in 2020, Abbasinezhad-Mood et al. [16] proposed an AKA protocol for secure group applications, addressing some of the above-mentioned issues, such as ESL attacks and private key leakage. Despite these improvements, their scheme allows a trusted authority (TA) to impersonate a smart meter and establish SKs with service provider (SP) [17]. Additionally, this protocol requires significant computational and communication resources due to its need for bilinear pairing computations. In 2021, Srinivas et al. [18] developed an anonymous AKA scheme using Schnorr’s signature. However, Baruah et al. [17] later revealed that this protocol is vulnerable to “man-in-the-middle” (MITM) and “impersonation attacks”. Further crypt-analysis showed that it also suffers from “key escrow” (KE) issues and ESL attacks. Yang et al. [19] showed that Shen et al.’s scheme [20] is insecure against MITM and “key compromise impersonation” (KCI) attacks and fails to ensure “perfect forward secrecy” (PFS), subsequently proposing an enhanced cloud-based solution. However, this enhanced scheme also faces KE problems and does not provide user anonymity. Chaudhry et al. [21] proposed an AKA scheme for secure group applications using ECC and symmetric encryption; however, this scheme also suffers from KE issues and is vulnerable to MITM attacks. Hajian et al. [22] assessed the shortcomings of four existing AKA schemes and proposed an improved device-to-device AKA scheme for IoT. However, this improved scheme is still vulnerable to MITM and KCI attacks and fails to provide PFS. In 2023, Chen et al. [23] developed an AKA scheme tailored for industrial control systems, but it incurs high operation and network costs, is vulnerable to ESL attacks, and cannot offer PFS. Rajkumar et al. [24] proposed an ECC-based certificateless signature aggregation scheme for vehicular ad hoc networks. Hu et al. [8] recently proposed an ECC-based AKA scheme for IoT environments, but it does not resist “physical capture attacks”.

To resolve these challenges, researchers have been developing physically secure AKA schemes for various environments. In 2023, Ma et al. [25] proposed a “physical-unclonable-function” (PUF)-based AKA scheme for smart grids that enables user anonymity. In 2024, Yu and Park [26] introduced a robust, anonymous AKA scheme using PUFs for vehicle-to-grid (V2G) networks, whereas Awais et al. [27] proposed a physically robust AKA scheme for vehicular ad hoc networks. Building on these advancements, we present suggestions that incorporate efficient, physically robust ways to enhance security in IoT environments. A comparative summary of previous works and the abbreviations used in this paper are defined in Table 1 and Table 2, respectively.

## 3. Preliminaries

### 3.1. Threat Model

In the previous work, the security analysis is conducted using the “extended Canetti and Krawczyk (eCK) model” [28]. This “threat model” allows an adversary to remove, modify, inject, and access all messages exchanged among the communicating entities. Moreover, the attacker can compromise and extract information from any number of devices. Through such captures, the attacker can retrieve secret information stored in the IoT node’s memory using “power analysis attacks” [29,30], as these devices typically lack physical security measures [8]. We conducted a security analysis using the same threat models as those employed by the previous work [8].

### 3.2. Elliptic Curve Cryptosystem (ECC)

Let q>3 represent a large prime and let E(a,b) be a nonsingular elliptic curve defined over the finite field $F_{q}$. Let *P* denote a generator point on this curve. The operation of group is the standard point addition on E(a,b), and *G* is a subgroup of order *p*, where p>q. Therefore, the following holds:

**Definition** **1.** *Elliptic curve decisional Diffie–Hellman problem (ECDDHP): Given three elliptic curve points xP, yP, and zP on $E_{p}$, determine whether zP equals the product xyP or is a random value.*

**Definition** **2.** Elliptic curve discrete logarithm problem (ECDLP): Given two points X,Y∈P, with X=xP, where $x\in Z_{p}^{*}$, find x.

### 3.3. Physical Unclonable Function

PUF [31] is a powerful solution for safeguarding resource-constrained smart devices against various types of security attacks. It produces unique outputs, similar to fingerprints, which are determined by the device’s distinct physical characteristics. Since PUF does not store secret keys, it is extremely difficult to replicate. This makes PUF particularly valuable for securing IoT devices, as it defends against threats like tampering, cloning, and side-channel attacks. The outputs are generated by nanoscale irregularities in the device’s manufacturing process, meaning that any modification made to the device will change its output. PUF is used to verify the authenticity of a device before establishing a secure session key. The procedures of PUF operation are outlined below:

(1) A random input challenge *C* is provided to the PUF.
(2) The challenge *C* interacts with the unique physical attributes of the device.
(3) The PUF generates a corresponding response *R* based on these attributes.
(4) The response *R* is then verified to authenticate the device and ensure secure communication.

## 4. Review of Hu et al.’s Scheme [8]

This section provides a concise overview of the previous [8] work and a threat model used for its crypt-analysis. The previous scheme is divided into three phases: initialization, registration, and AKA. Table 3 presents the relevant notations.

### 4.1. Initialization Phase

In this phase, the TA chooses an E(x,y) over the $F_{q}$ and its base point *P*; then, the TA selects “a collision-resistant hash function” h(). Finally, the TA broadcasts {E(x,y),q,P,h()} to open networks.

### 4.2. Registration Phase

This section presents the registration phase of the previous scheme, and its detailed steps are shown below.

(1) The service provider (SP) selects a random number $r_{sp}\in Z_{q}^{*}$ and an identity $ID_{sp}$. Then, the SP computes $R_{sp}=r_{sp}\cdot P$ and sends $\left\{ID_{sp},R_{sp}\right\}$ to the TA.
(2) The TA chooses a random number $r_{ta}^{sp}\in Z_{q}^{*}$ and computes the public key of the SP $PK_{sp}=R_{sp}+r_{ta}^{sp}$. Afterward, the TA sends $\left\{PK_{sp},r_{ta}^{sp},ID_{s},PK_{s}\right\}$ to the SP through a secure channel.
(3) The SP computes the private key $k_{sp}=\left(\left(r_{sp}+r_{ta}^{sp}\right)\right)\cdot P$ using $r_{ta}^{sp}$ and then checks whether $PK_{sp}\overset{?}{=}k_{sp}\cdot P$. If this is correct, then SP generates $WS_{s}=k_{sp}\cdot PK_{s}$ and stores $\left\{ID_{sp},k_{sp},ID_{s},WS_{s}\right\}$ in a memory. The end device (S) also stores $\left\{ID_{s},k_{s},ID_{sp},WS_{sp}\right\}$ through the same registration procedures.

### 4.3. AKA Phase

In this phase, the S and SP perform AKA for future communications, as detailed below.

(1) The S first selects a random number $x_{s}\in Z_{q}^{*}$ and generates a timestamp $T_{s}$. Then, the S computes $A_{s}=(x_{s}k_{x}$ mod q)·P and $B_{s}=x_{s}\cdot WS_{sp}$. The S also computes $EID_{s}=ID_{s}⊕B_{s}$ and $V_{s}=h(WS_{sp}||TS_{s}||DS_{s}\left|\right|B_{s})$, and it sends $\left\{A_{s},EID_{s},T_{s},V_{s}\right\}$ to the SP.
(2) The SP verifies the freshness of timestamp $T_{s}$. The S calculates $B_{sp}=k_{sk}\cdot A_{s}$ and $ID_{s}=EID_{s}⊕B_{sp}$, computes $V_{s}^{*}=h(WS_{sp}||TS_{s}||DS_{s}\left|\right|B_{s})$, and checks $V_{s}^{*}\overset{?}{=}V_{s}$.
(3) If the equality is verified, then SP chooses a random number $x_{sp}$ and a timestamp $T_{sp}$, and the SP generates $A_{sp}=(x_{sp}k_{sp}$ mod q)·P, $C_{sp}=x_{sp}\cdot B_{sp}$, the session key $SSK_{sp}=h(ID_{s}||ID_{sp}\left|\right|C_{sp})$, and $V_{sp}=h(WS_{s}\left|\right|T_{sp}||ID_{sp}||SSK_{sp})$. Finally, the SP sends $\left\{A_{sp},T_{sp},V_{sp}\right\}$ to the S.
(4) The S verifies the freshness of timestamp $T_{sp}$ and then computes $C_{s}=(x_{s}k_{s}$ mod $q)\cdot A_{sp}$, the session key $SSK_{s}=h(ID_{s}||ID_{sp}\left|\right|B_{s}\left|\right|C_{s})$, and $V_{sp}^{*}=(WS_{sp}\left|\right|T_{sp}||ID_{sp}||SSK_{s})$. Finally, the S checks whether $V_{sp}^{*}\overset{?}{=}V_{sp}$. If this is correct, then the SP and S successfully authenticate each other.

## 5. Security Weaknesses of Hu et al. Scheme [8]

In this section, we show that the previous scheme is vulnerable to “physical capture” and “impersonation attacks”. Additionally, we show that the scheme fails to secure session keys and authentication between entities.

### 5.1. Formal Security Analysis Using ROR Model

We demonstrate that the previous scheme does not guarantee session key security using mathematical analysis through the ROR model [9], a widely accepted method for formal security verification [10,11]. To assess protocol security, we first present the basics of the ROR model and use it to evaluate the security of the previous protocol.

- Participants: The protocol instances for the S and the SP are denoted as $\Pi_{S}^{inst_{1}}$ and $\Pi_{SP}^{inst_{2}}$, respectively.
- Accepted state: Once the message exchange process is completed, the oracle $\Pi^{inst}$ transitions to an accepted state, where its session identifier sid is defined by the sequence of all messages exchanged during the interaction.
- Partnering: Two instances, $\Pi_{S}^{inst_{1}}$ and $\Pi_{SP}^{inst_{2}}$, are considered partners if they share the same sid, reach the accepted state, and successfully complete the AKA procedure.
- Freshness: The instances ($\Pi_{S}^{inst_{1}}$, $\Pi_{SP}^{inst_{2}}$) are regarded as fresh if the session key exchanged between the S and SP remains secure and unexposed to the adversary *A*.
- Adversary: In the threat model of the previous protocol [8], an adversary *A* can fully manage the networks and use the ROR queries outlined in Table 4 to attempt to breach the protocol security.
- Semantic security: The adversary *A* seeks to extract the session key of an instance by manipulating an arbitrary nonce. Initially, *A* makes a guess about a bit *c* using ROR queries. If *A* accurately predicts the bit *c*, it wins, thereby undermining the semantic security of the protocol. The event where *A* wins is denoted as Win, and the session-key-breaking advantage for the previous protocol is expressed as $Adv_{P}=\left|2Pr\left[Win\right]-1\right|$.
- Random oracle: Every participant in the protocol is granted access to a random oracle, which is realized through a secure hash function denoted as *H*.

Through Definitions 1 and 2 and Theorem 1, we prove that the previous scheme does not ensure SK security.

**Theorem** **1.** *Within the threat model, we define $q_{h}$ as the number of queries made to the random oracle and Hash as the output length of H. Assuming that the adversary A, running within a polynomial time (t) against the previous protocol P, has an advantage $Adv_{P}^{ECDDHP}\left(t\right)$ in breaking the security of the session key,*

(1) $$\begin{array}{c} Adv_{P}^{ECDDHP}\geq \frac{q_{h}^{2}}{\left|Hash\right|}+2Adv^{ECDDHP}\left(t\right). \end{array}$$

**Table 4:** Queries of ROR model.

Queries	Explanations
$Execute(\Pi_{S}^{inst_{1}},$ $\Pi_{SP}^{inst_{2}})$	This query models an eavesdropping scenario, in which the adversary *A* can intercept and observe communications occurring over the open network.
$CorruptS(\Pi_{S}^{inst_{1}})$	This query simulates a “physical capture attack”, enabling the adversary *A* to retrieve data from the S.
$Send(\Pi^{inst},M)$	This query represents an active attack simulation, where the adversary *A* is able to send a message to the oracle $\Pi^{inst}$ and obtain a response in return.
$Test(\Pi^{inst})$	This query tests the freshness security of SK by presenting an arbitrary choice *c*; *A* is given either the actual SK (c=1) or an arbitrary value (c=0), or NULL (⊥) if it is not fresh.

This formal proof is executed around several games $G_{i}\left(i=0,1,2\right)$, where Win is the occurrence of *A* winning a game $G_{i}$.

- Game $G_{0}$: This game represents an active adversary scenario where *A* targets the scheme with a randomly chosen *c* at the start of the game and has a particular winning advantage.
  (2) $$Adv_{P}^{ECDDHP}=\left|2.Pr\left[Win_{0}\right]-1\right|$$
- Game $G_{1}$: This game depicts an “eavesdropping attack” in which the adversary *A* can monitor the transmitted messages through Execute queries and then performs a Test query to differentiate between the real $SSK_{s/sp}$ and a random value. In their scheme, $SSK_{s/sp}$ is derived as $SSK_{sp}=h(ID_{s}||ID_{sp}\left|\right|B_{sp}\left|\right|C_{sp})$ and $SSK_{s}=h(ID_{s}||ID_{sp}\left|\right|B_{s}\left|\right|C_{s})$. Despite accessing all public channel communications, *A* cannot compute $SSK_{s/sp}$, thereby failing to enhance its probability of winning the game. Then,
  (3) $$Pr\left[Win_{1}\right]=Pr\left[Win_{0}\right].$$
- This game simulates an impersonation attack where adversary *A* impersonates a legitimate S using $Send(\Pi^{inst},M)$, $CorruptS(\Pi_{S}^{inst_{1}})$, and several Hash queries. According to the assumptions of the previous work, *A* first performs the $CorruptS(\Pi_{S}^{inst_{1}})$ query to retrieve $\left\{ID_{s},k_{s},ID_{sp},WS_{sp}\right\}$ from the S memory. *A* can compromise $SSK_{s}$ using the real identity $ID_{s/sp}$ and $k_{s}$ without needing to solve the ECDLP or ECDDHP. Consequently, games $G_{1}$ and $G_{2}$ can be distinguished. Then,
  (4) $$\begin{array}{c} Adv_{P}^{ECDDHP}\geq \frac{q_{h}^{2}}{2|Hash|}. \end{array}$$

Upon completion of all games ($G_{0},G_{1},G_{2})$, *A* tries to guess the *c* correctly using the Test query. Therefore,

(5) $$\begin{array}{c} Adv_{P,G_{2}}^{A}=\frac{1}{2}. \end{array}$$

The result can be determined using Equations (2), (3) and (5).

(6) $$\begin{array}{ccc} \frac{1}{2}.Adv_{P}^{A} & = & |Pr\left[Win_{0}\right]-\frac{1}{2}|\\ & = & |Pr\left[Win_{1}\right]-\frac{1}{2}|\\ & = & |Pr\left[Win_{1}\right]-Pr\left[Win_{2}\right]| \end{array}$$

The final result is obtained using Equations (4)–(6).

(7) $$\begin{array}{c} Adv_{P}^{ECDDHP}\geq \frac{q_{h}^{2}}{\left|Hash\right|}+2Adv^{ECDDHP}\left(t\right) \end{array}$$

Finally, we eliminate the term $2Adv^{ECDDHP}\left(t\right)$ in Equation (7), as the security of the session key can be compromised without needing to solve the ECDDHP and ECDLP. This formal proof demonstrates that it fails to secure session keys.

### 5.2. Informal Security Analysis

In this section, we perform logical security analysis to prove that the previous scheme cannot withstand “physical capture and impersonation attacks”.

#### 5.2.1. Physical Capture Attack

As discussed in Section 4.2, the TA stores the data $\left\{ID_{sp},k_{sp},ID_{s},WS_{s}\right\}$ in the S memory without using cryptographic techniques. Within the security analysis in the previous scheme [8], the authors considered physical capture attacks. Therefore, an adversary *A* can retrieve stored values through “power analysis attacks” [29,30]. This vulnerability allows *A* to execute impersonation attacks and breach user privacy.

#### 5.2.2. End Device Impersonation Attack

An impersonation attack occurs when an attacker masquerades as a legitimate user to gain unauthorized benefits. The attack is considered successful if the attacker obtains secret parameters and makes AKA requests that are undetected by legitimate entities.

**Figure 1:**
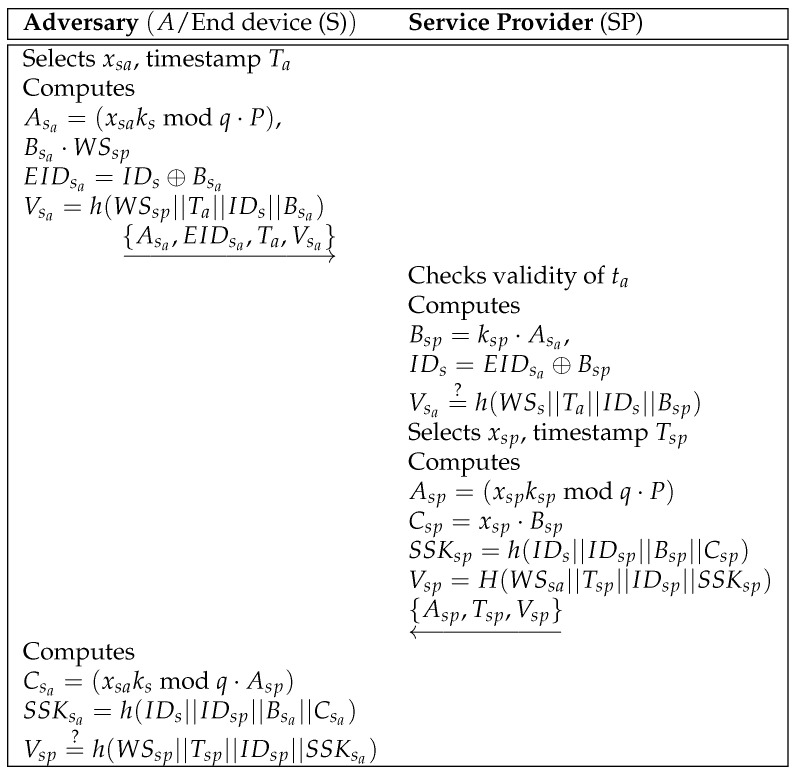
End device impersonation attack in the previous scheme.

In Hu et al.’s scheme [8], security is assessed using their proposed assumptions. However, by obtaining an end device and retrieving its stored data through physical capture attacks, *A* can easily compute a login request $\left\{A_{s},EID_{s},T_{s},V_{s}\right\}$ and calculate the correct session key $SSK_{s}=h(ID_{s}||ID_{sp}\left|\right|B_{s}\left|\right|C_{s})$. This vulnerability arises because the secret parameters stored in the S memory lack cryptographic protection. Therefore, the previous scheme fails to defend against end device impersonation attacks. Figure 1 shows a process affected by this issue.

#### 5.2.3. Insecure Mutual Authentication

According to Section 5.2.1 and Section 5.2.2, *A* can effortlessly masquerade as a legal vehicle to gain entry into the IoT networks described in the previous scheme and successfully authenticate with the participating nodes. *A* can also compromise the session key between an end device and the SP. Thus, it fails to achieve secure mutual AKA.

#### 5.2.4. Correctness of Threat Model

The authors asserted that the overall system remains secure because only a single device is captured in a physical capture attack. However, the capture of a medical device or another critical component of the system could present serious security risks. Therefore, a method that guarantees the security of all end devices against physical capture attacks should be developed. We propose three solutions (1–3) to enhance security and privacy in Section 6.

### 5.3. AVISPA: Simulation Analysis

**Figure 2:**
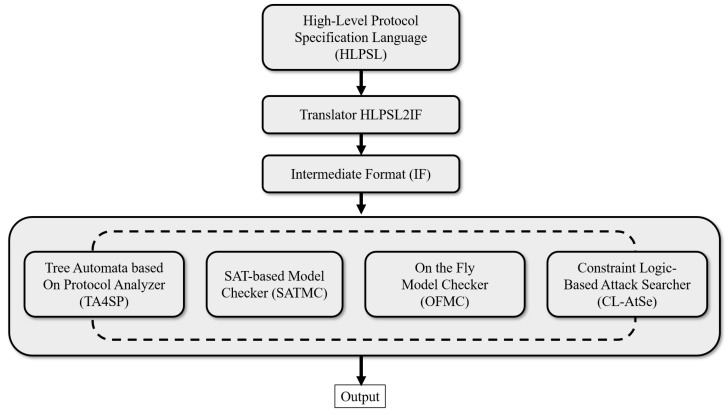
AVISPA simulation procedure.

The security of cryptographic protocols is rigorously verified using AVISPA, a prominent tool for evaluating security schemes [12,13]. AVISPA checks schemes for vulnerabilities, such as replay and MITM attacks. It uses the “high-level protocol specification language” (HLPSL) [32] to specify the security aspects of protocols. This tool leverages four back-end models [33]: the “constraint-logic-based attack searcher” (CL-AtSE), “on-the-fly model checker” (OFMC), “SAT-based model checker” (SATMC), and “tree-automata-based protocol analyzer” (TA4SP). The HLPSL code is converted into an intermediate format with the HLPSL2IF translator, which is then evaluated using these back-end models to determine the protocol security properties. Figure 2 depicts this workflow, and further details on HLPSL are provided in [13,32].

#### 5.3.1. AVISPA Simulation Environments

The simulation was carried out on a machine running Ubuntu 10.10, featuring 2 GB of RAM, and driven by an Intel Core i9-11900K processor operating at 3.50 GHz, with a total of 64 GB of RAM.

#### 5.3.2. HLPLS Specifications

**Figure 3:**
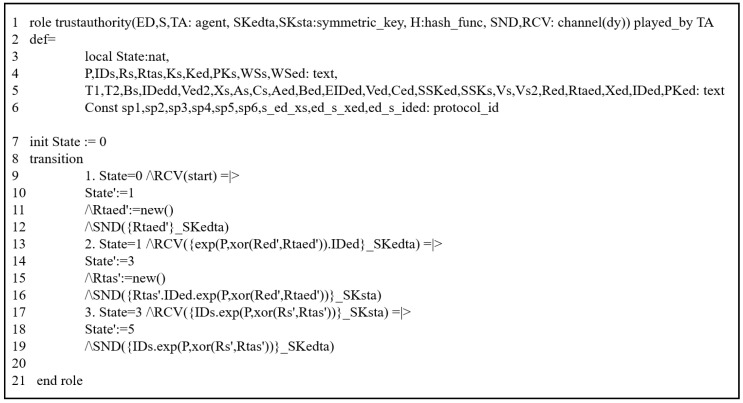
HLPSL description: TA’s role.

**Figure 4:**
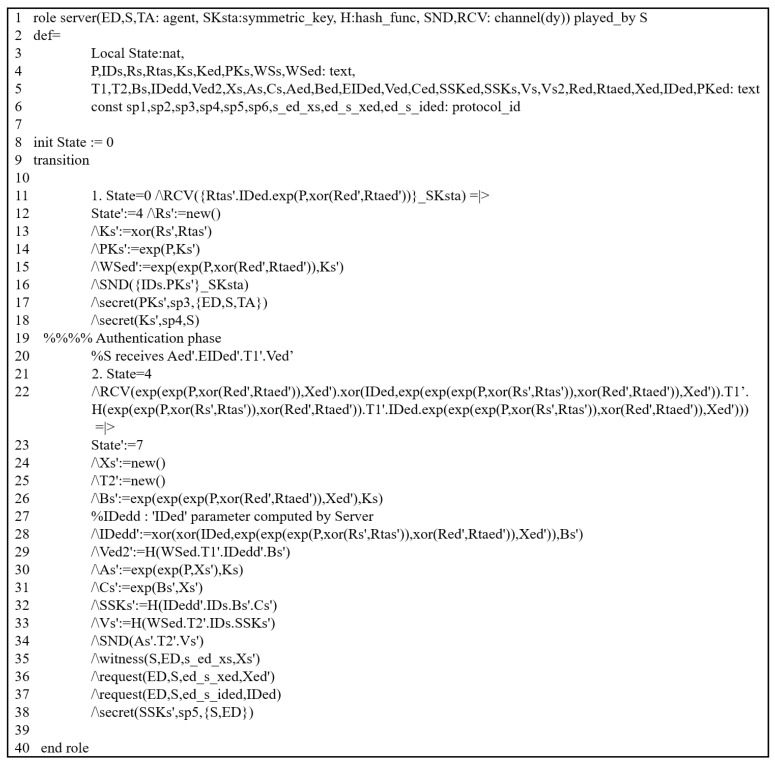
HLPSL description: SP’s role.

**Figure 5:**
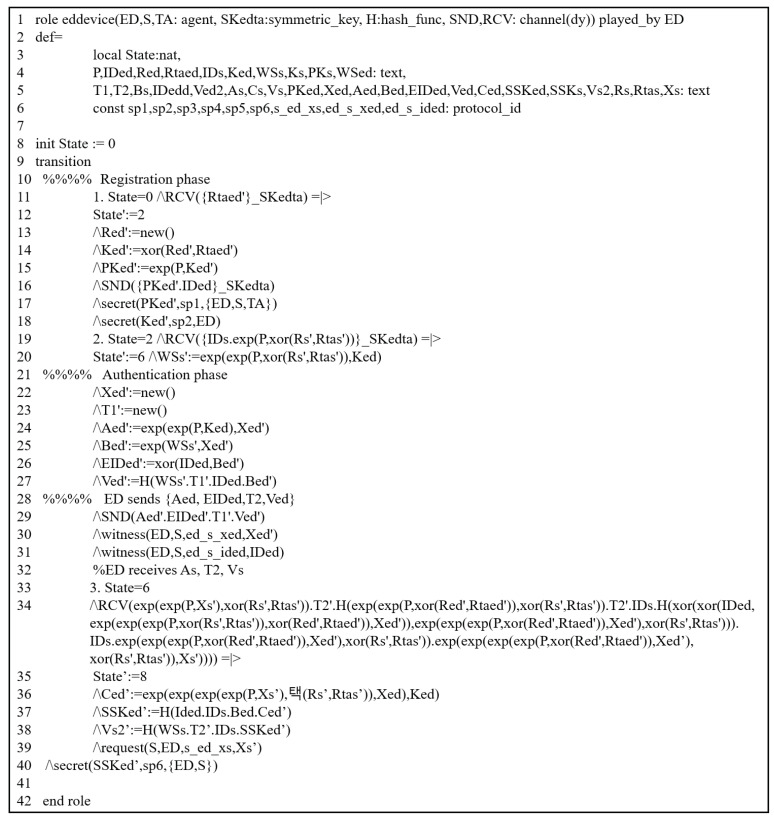
HLPSL description: end device’s role.

**Figure 6:**
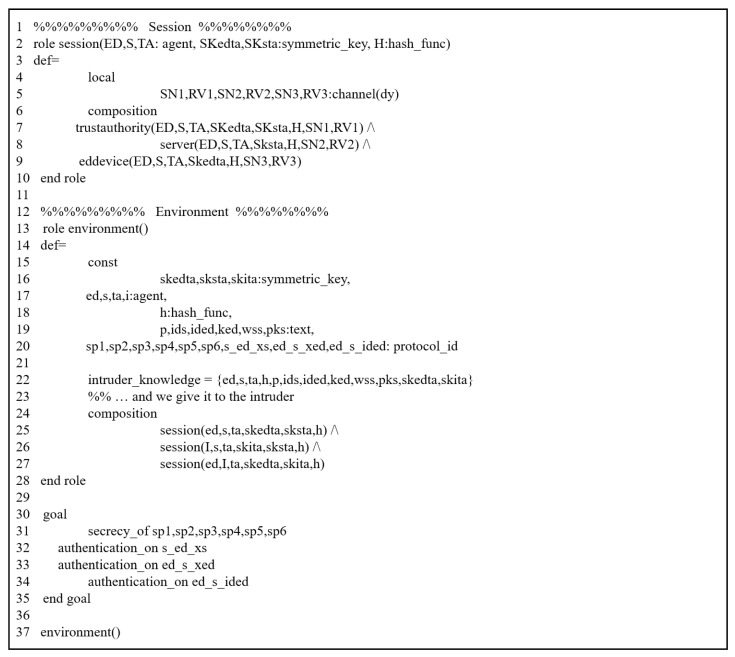
HLPSL description: session and environment.

The previous scheme was assessed through this simulation by modeling all phases of their scheme using HLPSL codes. We evaluated it by analyzing the authentication procedures between the involved entities. It includes three primary roles: trusted third party (TA), service provider (SP), and end device (S). The HLPSL codes of these entities are depicted in Figure 3 (TA), Figure 4 (SP) and Figure 5 (S). The environment and session details are illustrated in Figure 6.

#### 5.3.3. Simulation Results

To demonstrate that the previous scheme is susceptible to replay and MIMT attacks, we used OMFC and CL-AtSe using the provided HLPSL codes (Figure 3, Figure 4, Figure 5 and Figure 6).

**Figure A1:**
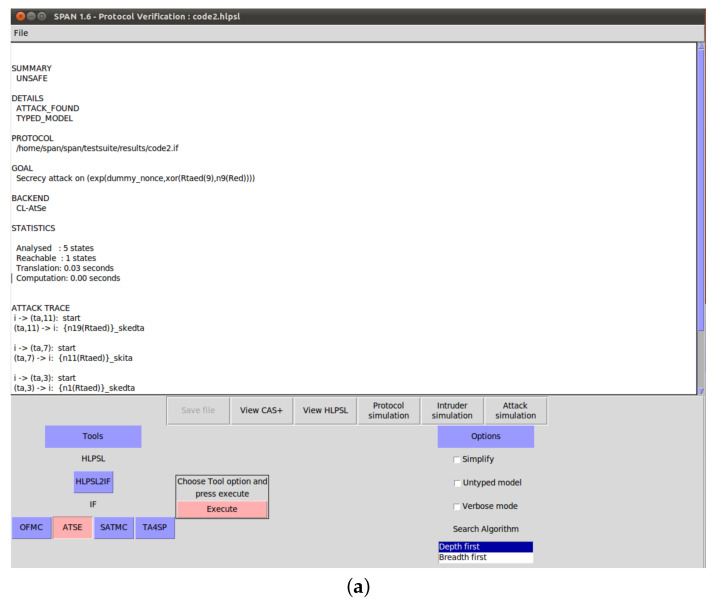

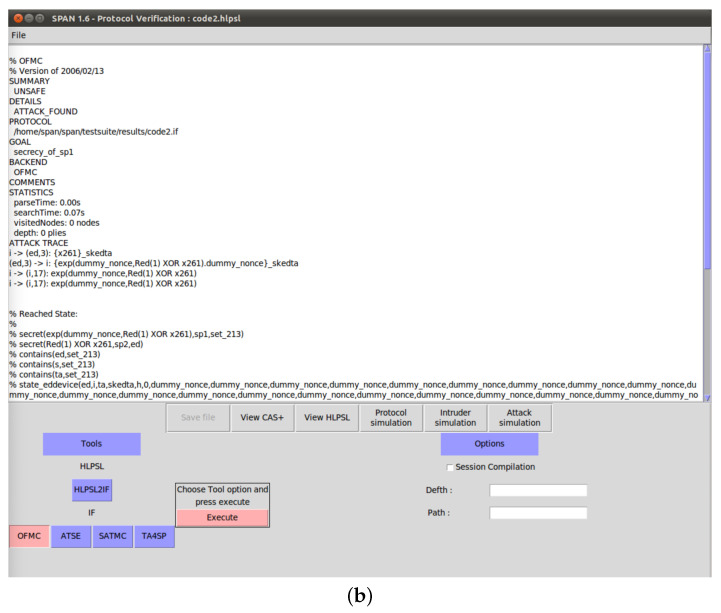
Simulation results. (**a**) Result: CL-AtSe. (**b**) Result: OFMC.

- OFMC: The total search time was 0.07 s, and the reached states are described in Figure A1 in Appendix A.
- CL-AtSe: For CL-AtSe, the translation time was 0.03 s, during which five states were analyzed.

The OFMC and CL-AtSe results in Figure A1 indicate an “unsafe” status. Hence, the previous scheme failed to thwart replay and MIMT attacks.

## 6. Security Fixes

The previous scheme [8] has notable security flaws, primarily due to the storage of secret information in end devices without cryptographic techniques. These flaws allow an attacker to access the private data in the memory of devices easily, enabling them to masquerade as legitimate entities and compromise the session keys between participating nodes. These critical security weaknesses are detailed in Section 5.

Many AKA schemes proposed in recent decades involve storing secret data in device memory for authentication between communicating entities. Hu et al. [8] claimed that the overall system remains secure in their scheme because only one device is captured through a physical capture attack. However, the capture of medical devices or devices critical to the system could pose significant security risks. Therefore, a method that secures all end devices against physical capture attacks should be designed. To address the security problems identified in a previous protocol, we propose the following key guidelines.

Sol. 1. According to Section 4.2, the TA avoids storing secret data $\left\{ID_{s},k_{s}\right\}$ as plaintext to resist “physical capture attacks”. According to their security analysis, an attacker can easily compromise the memory of end devices and obtain their data. Thus, end devices should securely store these data using masked techniques, such as exclusive OR (XOR) operations combined with a hash function. For example, instead of directly storing $ID_{s}$ and $k_{s}$ on an end device, these values can be stored by performing an XOR operation with certain parameters, such as $ID_{V}⊕h(ID_{V}\left||secret\_parameter\right)$ and $K_{s}⊕h(ID_{v}||h(ID_{V}\left||secret\_parameter)\right)$, or by generating values based on the hash of $ID_{s}$, like $PID_{s}=h(ID_{s},random\_number,h(sec$-$ret\_parameter||ID_{s}))$. This solution can prevent the adversary from easily obtaining $\left\{ID_{s},k_{s}\right\}$, as these values are not stored in the memory as plaintext.
Sol. 2. The use of PUFs on end devices is recommended to resist physical capture attacks. In a previous scheme, the identity of an end device $ID_{s}$ and a secret parameter $k_{s}$ can be easily extracted, potentially facilitating impersonation attacks. However, a PUF-based AKA scheme protects against physical capture attacks by generating unique secret parameters using a physical semiconductor structure [26,27]. For example, the session key can be computed using unique random values produced by a PUF. The user sends their challenge *C* to the PUF, which then generates the corresponding response *R*. Since the *R* is based on the PUF’s physical characteristics, it cannot be reproduced by any other device. Therefore, this response can be used in the key establishment process to ensure security. In a previous scheme, the adversary can easily generate the session key $SSK_{s}$ by knowing the secret values $ID_{s}$ and $k_{s}$. Therefore, by incorporating the h(PUF) value into session key generation ($SSK_{s}=h((PUF||ID_{s})||ID_{sp}\left|\right|B_{s}\left|\right|C_{s})$), an adversary cannot compromise the session key without breaking the difficulty of the PUF. This suggestion resolves the above-mentioned security weaknesses and other potential flaws.
Sol. 3. In a previous scheme, end devices rely on one-factor authentication during AKA, which does not require any user input. This mechanism makes physically compromised devices susceptible to attacks. This vulnerability should be mitigated by adopting a two-factor authentication (2FA) or three-factor authentication (3FA) mechanism for end devices, particularly when sessions are refreshed. A 2FA system uses either passwords or biometric data. For enhanced security, a 3FA system can be used, integrating both password and biometric verification. This additional security measure greatly enhances protection against unauthorized access. We recommend implementing a 3FA mechanism incorporating biometric verification through a fuzzy extractor [34] combined with PUF-based secret parameters. A “fuzzy extractor”, which is frequently used in biometric authentication, can be adopted to develop a resilient 3FA-based AKA scheme. In this enhanced mechanism, the security of secret parameters can be reinforced using masking techniques, such as XOR and hash functions, in conjunction with the biometric data generated by the “fuzzy extractor”. Consequently, even if end devices are physically compromised by an adversary, the secret parameters used in the AKA process remain protected and inaccessible without the corresponding biometric data.

The proposed solutions are intended to reduce the risk of impersonation attacks on end devices; they do not address all possible security vulnerabilities. Nonetheless, these improvements significantly enhance system security and raise the difficulty level for potential attackers. The times required to perform XOR and PUF operations on low-specification Arduino devices are 0.95 and 1.22 ms, respectively, indicating minimal computational requirements and demonstrating effective operation on even very small devices. Moreover, by incorporating a PUF module into low-spec devices for key generation, it is possible to implement 3FA without relying on biometrics, thereby enhancing the overall security level. Therefore, the proposed solutions are highly applicable and efficient in numerous IoT environments with low-specification devices [27].

The authors of the previous work exerted considerable effort in designing an IoT AKA scheme. However, a more comprehensive examination of their scheme from various angles would have been beneficial. The progression of research in this field involves various approaches from different studies. The current paper underscores the continuing need for the development of robust, efficient AKA schemes in IoT environments.

## 7. Conclusions and Future Works

This paper refers to the recent secure ECC-based IoT AKA scheme. We prove that this scheme does not prevent impersonation attacks on end devices, thereby not securing AKA and satisfying the security properties under their security assumptions. By applying formal mathematical analysis with the ROR model, we show that the scheme does not provide sufficient security for the session key. In addition, we perform an AVISPA simulation, a well-established tool for formal verification, to assess the protocol’s vulnerabilities. These identified security flaws render the protocol impractical for real-world implementation. To address these issues, we suggest improvements to enhance security and design a more reliable and efficient AKA scheme tailored for IoT environments. Our future work will involve developing a blockchain-based AKA scheme that is adaptable to various settings with resource-limited devices (Arduino, Raspberry Pi, ARM Boards, etc.), incorporating the proposed solutions. Additionally, given the significance of physical capture attacks in distributed systems, we plan to explore distributed authentication approaches. A key challenge in this area is establishing a secure connection between different types of embedded devices and the PUF module.

## Figures and Tables

**Table 1:** A comparative summary of existing AKA scheme for IoT.

Scheme	Year	Cryptographic Primitives	Advantages/ Description	Shortcomings/ Limitations
Feng et al. [14]	2023	ECC hash function	Efficient AKA scheme for heterogeneous IoT networks Provide lightweight computation & communication overheads	Does not resist ESL attacks Does not resist physical capture attacks
Srinivas et al. [18]	2024	ECC Schnorr’s Signature hash function	ECC-enabled Schnorr’s signature-based AKA scheme for smart grid Provide the procedures of dynamic node addition	Does not resist impersonation attacks Does not resist MITM attacks Does not resist physical capture attacks
Chaudhry et al. [21]	2022	ECC hash function	Efficient AKA scheme for smart grid infrastructure Provide user anonymity	Does not resist MITM attacks Does not resist key escrow attacks Does not resist physical capture attacks
Hajian et al. [22]	2022	ECC hash function	Efficient device to device AKA scheme for IoT Provide key update procedures Perform the formal simulation analysis	Does not resist MITM attacks Does not resist impersonation attacks Does not resist physical capture attacks
Rajkumar et al. [24]	2024	ECC hash function	ECC-based certificate-less aggregation scheme in vehicular IoT. Provide lightweight certificate-less authentication Provide batch verification	Does not resist impersonation attacks Does not resist physical capture attacks
Hu et al. [8]	2024	One-way hash function Physical unclonable function	ECC-based AKA scheme for IoT networks Perform the formal simulation analysis	Does not resist impersonation attack Does not resist MITM attack Does not resist physical capture attacks

**Table 2:** Abbreviations.

Notations	Descriptions
IoT	Internet of Things
AKA	Authentication and key agreement
ESL	Ephemeral secret leakage
MITM	Man in the middle
KCI	Key compromise impersonation
ECC	elliptic curve cryptosystem
PFS	Perfect forward secrecy
PUFs	Physical unclonable functions
ECDLP	Elliptic curve discrete logarithm problem
ECDDHP	Elliptic curve decisional Diffie–Hellman problem
HLPSL	High-level protocol specification language

**Table 3:** Notations used in this work.

Notation	Description
TA, KGC	A trusted authority, a key generation center
S, SP	End device and server
$SP_{j},ID_{SP_{j}}/SM_{i},ID_{SM_{i}}$	*j*th/*i*th service provider/smart meter and its identities
$E_{q}\left(x,y\right)$	A non-singular elliptic curve
P	A base point of elliptic curve $E_{q}\left(x,y\right)$
$t,T_{pub}$	Private and public key pairs of TA
$SSK_{i/j}$	The session key
$A_{V},B_{R},X_{V}$	Authentication parameters
⊕,||	The XOR and concatenation operations
*T*	A timestamp
h()	The collision resistant hash function

## Data Availability

Data are contained within the article.
